# An Atypical Presentation of Fulminant Myocarditis Secondary to COVID-19 Infection

**DOI:** 10.7759/cureus.9179

**Published:** 2020-07-14

**Authors:** Ivan Richard, Bracha Robinson, Amanda Dawson, Ashley Aya, Rana Ali

**Affiliations:** 1 Internal Medicine, Hackensack Meridian Ocean Medical Center, Brick, USA

**Keywords:** fulminant myocarditis, covid 19

## Abstract

The potential etiologies of fulminant myocarditis include autoimmune diseases, infections, drug hypersensitivity, and drug/toxin reactions. We present an atypical case of fulminant myocarditis in a patient with a history of diabetic ketoacidosis with recent novel coronavirus (COVID-19) infection, who presented with acute upper gastrointestinal bleeding. The patient improved with a three-day course of methylprednisolone 1 gram daily.

## Introduction

When a young adult with a history of type 1 diabetes mellitus and multiple episodes of diabetic ketoacidosis (DKA) presents with a suspected acute upper gastrointestinal bleed and a confirmed infection with coronavirus (COVID-19), the potential complications are numerous. While fever and cough have been found to be the most common symptoms, it is theorized that cardiac pathology is a late manifestation of COVID-19 infection [1,2]. The theory explains that cardiac manifestations are a result of the virus propagating in and circulating from the respiratory tract to various other organ systems through the blood or lymphatic system or myocardial injury from an intense systemic inflammatory response [2]. We present this case to raise awareness about fulminant myocarditis as a complication of COVID-19, as well as its possible atypical presentations. We also hope to shine a spotlight on the need for future studies to determine whether the main cause of damage to the heart in fulminant myocarditis is COVID-19, as well as its most effective treatment.

## Case presentation

The patient is a 28-year-old Caucasian woman with a history of diabetes mellitus type 1, diabetic gastroparesis, asthma, anxiety, depression with multiple previous episodes of DKA, and recent COVID-19 infection who was brought to the emergency department after she was found to be lethargic and covered in coffee ground emesis at home. Although she denied smoking, alcohol, and substance use, her medical records did have a questionable history of IV drug use. On physical examination, her vital signs were as follows: blood pressure of 70/38 mmHg, pulse rate of 144 bpm, respiratory rate of 30 rpm, and peripheral capillary oxygen saturation (SpO^2^) of 90% on 15 liters of a nonrebreather mask, and she was afebrile. She was in acute respiratory distress, tachypneic, and lethargic. Breath sounds were clear to auscultation bilaterally. Heart sounds were tachycardic, with a regular rhythm, and no appreciable murmurs. Her abdomen was nondistended, soft, nontender, with a nonpalpable liver and spleen. The patient had no peripheral edema in the extremities, which were cool to touch with 1+ peripheral pulses bilaterally. There were no focal neurological deficits found on the physical examination.

**Figure 1:**
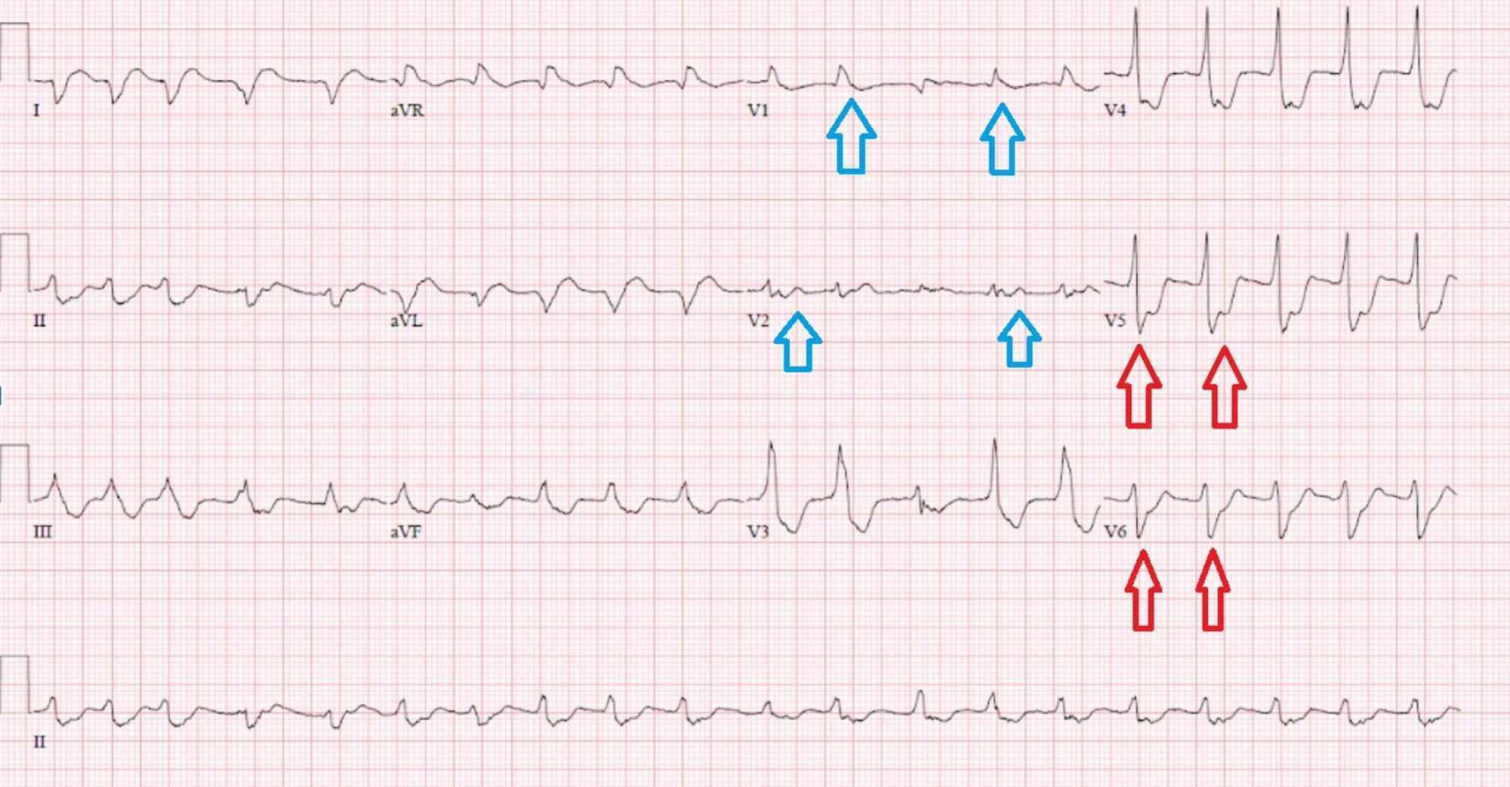
ECG demonstrating sinus tachycardia at 119 bpm with low voltage and nonspecific ST changes in the lateral leads (red arrows) and a possible septal infarct (blue arrows).

**Figure 2:**
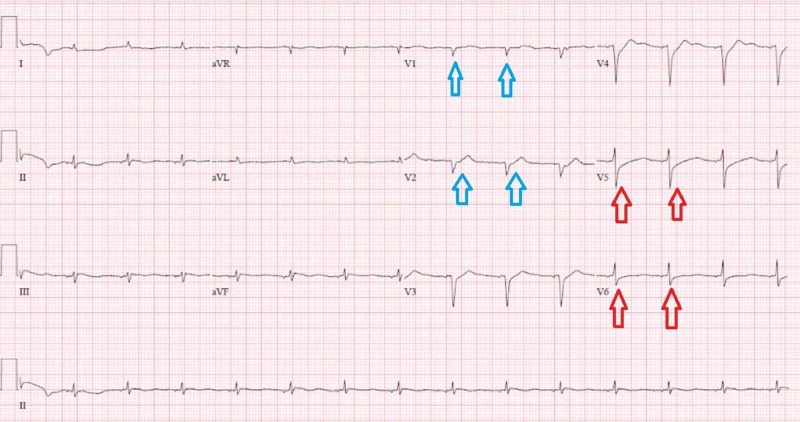
ECG demonstrating a sinus rhythm at 85 bpm with nonspecific ST changes in the lateral leads (red arrows) and a possible septal infarct (blue arrows).

Abnormal results of initial laboratory tests were as follows: white blood cell of 29 x 10^3^/uL (reference range: 4.5-11 x 10^3^/uL) hemoglobin of 10.3 g/dL (reference range: 12-16 g/dL), hematocrit of 31% (reference range: 35.0%-48.0%), creatinine of 4.4 mg/dL (reference range: 0.44-1.00 mg/dL), glucose of 1,679 mg/dL (reference range: 70-99 mg/dL), anion gap of 46 mmol/L (reference range: 5-13 mmol/L), carbon dioxide of 6 mmol/L (reference range: 24-31 mmol/L) , potassium of 2.9 mmol/L (reference range: 3.5-5.2 mmol/L), lactic acid level of 17.1 mg/dL (reference range: 0.5-2 mmol/L), C-reactive protein (CRP) of 2.47 mg/dL (reference range: 0.0-0.74 mg/dL), lactate dehydrogenase (LDH) of 296 U/L (reference range: 91-200 U/L), ferritin of 119 ng/mL (reference range: 11-307 ng/mL), and troponin of 0.04 ng/mL (reference range: <0.04 ng/mL). Arterial gas analysis post-intubation showed a pH of 7.17 (reference range: 7.35-7.45), carbon dioxide partial pressure of 12.9 mmHg (reference range: 35-50 mmHg), and oxygen partial pressure of 317 mmHg (reference range: 85-106 mmHg). Her urine toxicology screen was negative. The patient’s electrocardiogram (ECG) showed a wide complex tachycardia with a heart rate of 119 bpm and accelerated junctional rhythm. EKG was also significant for ST segment depression in the lateral leads and ST elevation in leads I and aVL, showing sinus tachycardia at 119 bpm with low voltage and nonspecific ST changes in the lateral leads, and possible septal infarct (Figure 1). Her chest X-ray findings were suspicious for COVID-19. The patient was intubated for airway protection and hypoxic respiratory failure and was started on norepinephrine for the maintenance of blood pressure. She was also started on an insulin drip for the management of DKA and broad-spectrum antibiotics coverage with IV vancomycin and piperacillin-tazobactam. The patient was then transferred to the intensive care unit and received IV potassium. Overnight the patient had an episode of ventricular tachycardia. An ECG at that time showed sinus rhythm with diffuse T-wave inversions and a new right bundle branch block. Her troponin I level eight hours after admission was 46 ng/mL (reference range: <0.04 ng/mL). An ECG performed at that time showed a sinus rhythm at 85 bpm with nonspecific ST changes in the lateral leads and a possible septal infarct (Figure 2).

**Figure 3:**
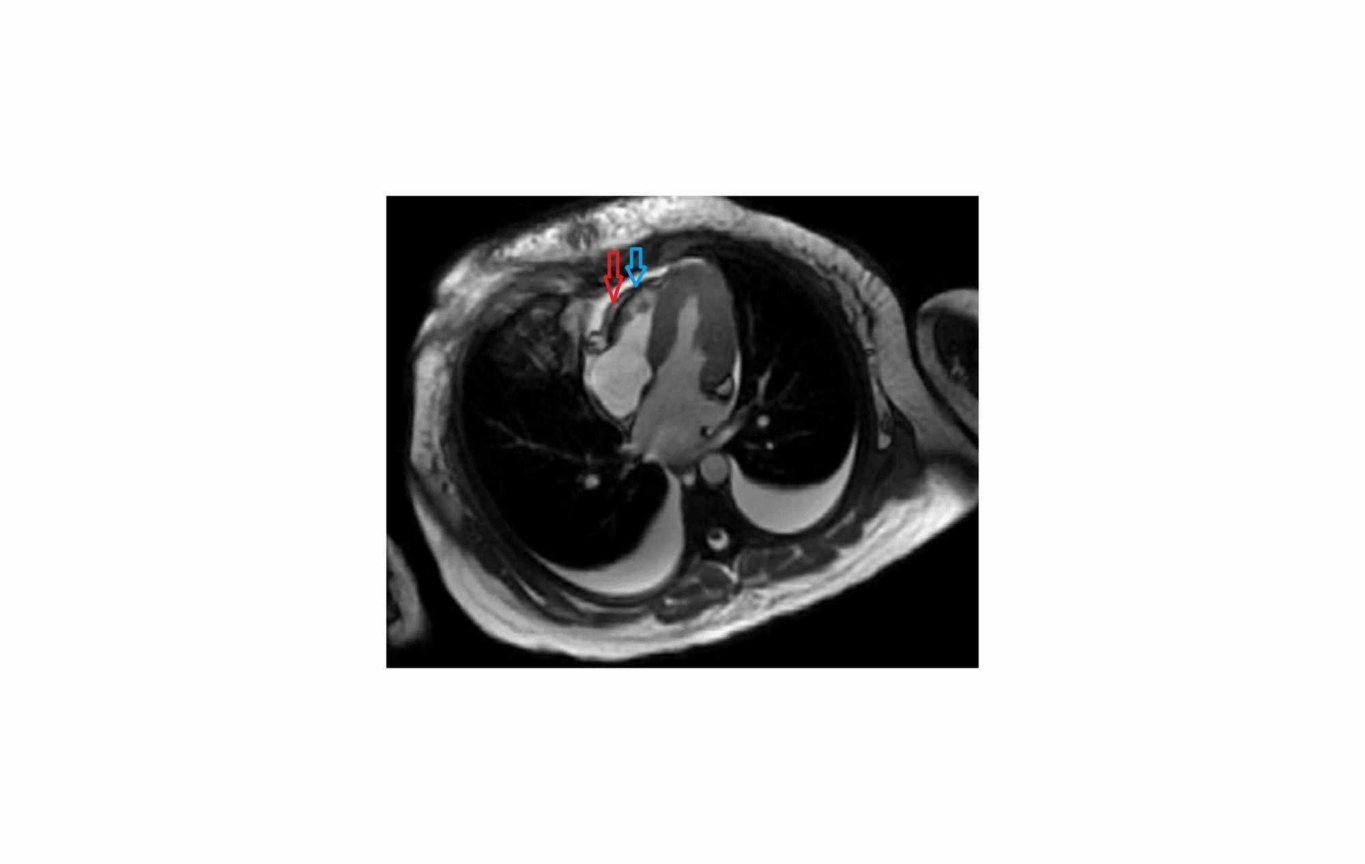
A four-chamber view of the patient’s cardiac MRI with contrast enhancement, demonstrating subepicardial myocardial fibrosis and necrosis in the basal to mid-anteroseptal and anterior walls (red arrow). There is hyperemia and myocardial edema in the mid-anteroseptum (blue arrow).

The cardiology service was consulted for non-ST segment elevation myocardial infarction, and they recommended both left and right heart catheterization with possible intervention. Prior to transferring the patient to the catheterization lab, a bedside echocardiogram was performed, which was significant for a left ventricular ejection fraction (LVEF) of 26-30% and mild mitral regurgitation. Once transferred, the patient’s left and right heart catheterization found normal coronary arteries but elevated filling pressures and a reduced cardiac index of 1.9 L/minute/m^2^ (reference range: 2.6-4.2 L/minute/m^2^). At that time, cardiogenic shock secondary to fulminant myocarditis from COVID-19 was suspected. An Impella device (ABIOMED Inc., Danvers, MA) was placed in the left ventricle to assist in heart pumping. The patient was started on IV dobutamine and heparin drips, and norepinephrine was stopped. She underwent a cardiac MRI that showed myocardial necrosis, fibrosis, and hyperemia, indicating myocarditis according to the Lake Louise criteria (Figure 3). Her anion gap closed, her insulin drip was stopped, and COVID-19 testing was negative. The patient’s hospital stay was complicated by acute oliguric renal failure, which improved with improvement in cardiac function. The patient’s COVID myocarditis was treated with pulse dose IV methylprednisolone 1 gram daily for three days. The patient clinically improved with IV steroids. An echocardiogram performed three days later, showed an LVEF of >55%, without significant valvular disease. The Impella device was then removed, dobutamine was weaned off, and the patient was extubated the next day.

## Discussion

A COVID-19 study from China, published in 2020, found that the most common symptoms of viral infection were fever, cough, fatigue, and shortness of breath [1]. Inciardi et al. described cardiac pathology as a late manifestation of COVID-19 respiratory infection [2]. Cardiac manifestations of COVID-19 have been hypothesized to be the result of the virus propagating in and circulating from the respiratory tract to various other organ systems through the blood or lymphatic system or myocardial injury from an intense systemic inflammatory response [2]. The underlying inflammatory process associated with COVID-19 can present either with clear symptoms, as previously mentioned, or subclinically with the etiology found only on autopsy [3]. Among the different etiologies of myocarditis, viral infections are one of the most common causes of infectious causes [4]. In patients with a recent history of influenza-like syndrome, who subsequently present with chest pain, in addition to laboratory, ECG, and echocardiogram findings suggestive of acute coronary syndrome with a coronary angiogram negative for obstructive disease, focal myocarditis should be suspected [5]. According to Yang et al., inflammatory cells have been found in the alveoli of COVID patients, thus justifying the use of steroids [6]. Some sources have hypothesized that COVID binds to viral receptors on the cardiac myocytes, which leads to replication of the viral capsid proteins and the viral genome [7,8].

Our patient was recently infected with COVID-19 and tested positive. She had a sudden onset of lethargy, gastrointestinal bleed, hemodynamic instability, renal insufficiency, hypoxemia, myocardial injury, and reduced myocardial contraction capability. All of the aforementioned are part of the diagnostic criteria for fulminant myocarditis [9]. The incidence of fulminant myocarditis appears to be low. Zeng et al. reported 419 COVID-19 cases, of which 32 had elevated troponin I, with only 2 of those patients found to have fulminant myocarditis [10]. That being said, other studies have confirmed the association between COVID infection and myocarditis [11]. According to Xu et al., heart tissue containing inflammatory infiltrates without viral inclusion bodies have been found in autopsies of COVID-19 patients [3]. Despite this, other studies have found that the viral load in COVID-19 patients is not the sole indicator of improvement in cardiac structure and function, leading to the suggestion that an immune response could be an important factor in myocarditis [12,13]. A potent immune response from hypoxia or viral infection of the myocytes could cause increased vascular permeability leading myocardial edema [14]. Tavazzi et al. found low-grade inflammation without myocyte necrosis in pathological studies of patients clinically diagnosed with severe myocarditis, suggesting that the effect on cardiac myocytes is either from a viremic phase or the transposition of infected alveolar macrophages [15]. Interestingly, they did not find cardiac myocytes with viral particles but did find non-specific changes, mainly focal myofibrillar lysis [15]. Other studies have found extrapulmonary distribution of COVID-19 RNA in the heart, kidneys, cerebrum, intestines, and lymph nodes [16,17]. Shakoory et al. promote the idea of specific anti-inflammatory treatments citing the survival benefit to patients in sepsis with hyper-inflammation who underwent trials of interleukin-1 antagonists [18]. No significant difference in mortality was reported in a 2013 Cochrane review of 8 randomized controlled trials that included 719 patients, observing the use of corticosteroids versus control groups for the treatment of viral myocarditis [19].

## Conclusions

In our case of fulminant myocarditis, we attributed the patient’s condition to viral infection with COVID-19 given her recent positive test prior to admission. We present this case to raise awareness of fulminant myocarditis as a complication of COVID-19, as well as its possible atypical presentations. A careful physical examination, diagnostic/laboratory testing, diagnostic cardiac intervention, and critical care management were crucial in this case. Future studies are needed to determine if the main cause of damage in fulminant myocarditis secondary to COVID-19 is due to direct viral infection or immunological mechanisms, as well as to assess the efficacy of the various anti-inflammatory treatments including corticosteroids, IV immunoglobulin, and interleukin-1 antagonists.
